# Analytical Study of the Effect of the System Geometry on Photon Sensitivity and Depth of Interaction of Positron Emission Mammography

**DOI:** 10.1155/2012/605076

**Published:** 2012-09-20

**Authors:** Pablo Aguiar, Cristina Lois

**Affiliations:** ^1^Nuclear Medicine Department (IDICHUS), 15706 Santiago de Compostela, Spain; ^2^Molecular Imaging Group (IDIS), 15706 Santiago de Compostela, Spain

## Abstract

Positron emission mammography (PEM) cameras are novel-dedicated PET systems optimized to image the breast. For these cameras it is essential to achieve an optimum trade-off between sensitivity and spatial resolution and therefore the main challenge for the novel cameras is to improve the sensitivity without degrading the spatial resolution. We carry out an analytical study of the effect of the different detector geometries on the photon sensitivity and the angle of incidence of the detected photons which is related to the DOI effect and therefore to the intrinsic spatial resolution. To this end, dual head detectors were compared to box and different polygon-detector configurations. Our results showed that higher sensitivity and uniformity were found for box and polygon-detector configurations compared to dual-head cameras. Thus, the optimal configuration in terms of sensitivity is a PEM scanner based on a polygon of twelve (dodecagon) or more detectors. We have shown that this configuration is clearly superior to dual-head detectors and slightly higher than box, octagon, and hexagon detectors. Nevertheless, DOI effects are increased for this configuration compared to dual head and box scanners and therefore an accurate compensation for this effect is required.

## 1. Introduction

Breast cancer is one of the most commonly diagnosed cancers and one of the leading causes of cancer related deaths in women [1]. Early detection of the disease can improve the treatment effectiveness and often also the patient's quality of life. A number of imaging techniques can be used to aid in the diagnosis and staging of breast cancer, being anatomical imaging techniques such as X-ray mammography, ultrasonography, and magnetic resonance imaging (MRI) the most employed [2–4]. However, these techniques are affected by two factors that limit their effectiveness: breast density and the woman's hormonal status [5, 6]. Because of these limitations, many women with suspicious breast masses have to undergo invasive breast biopsies for accurate diagnosis. 

Metabolical imaging techniques such as positron emission tomography (PET) are increasingly being used in oncology [7]. PET is not affected by the limiting factors mentioned above and it has been shown to be more accurate in differentiating cancerous and benign breast lesions than the anatomical techniques alone [8]. Thus, PET potentially translates into reduction in unnecessary breast biopsies, which could significantly lower costs associated with breast cancer detection and staging, and reduce patient trauma.

Positron emission mammography (PEM) cameras are novel-dedicated PET systems optimized to image the breast. By reconstructing the radiotracer distribution inside the breast, tomographic images of breast lesions are obtained in a noninvasive procedure. Compared to conventional whole-body PET systems, PEM cameras cover a smaller field of view that is limited to a single breast. The detectors are arranged around the breast so that their performance can be higher at a lower cost. The photon sensitivity of a PET system is related to the ratio between detected and emitted photons (i.e., detected counts and injected activity) and is mainly determined by the system geometry and the type and volume (thickness) of the detector. Thus, the sensitivity is increased in PEM cameras compared to conventional PET due to the proximity of the detectors to the breast. Due to this, gamma rays penetrate a significant distance into the detector before detection. This distance is called depth of interaction (DOI) and it can produce an uncertainty in the calculation of the photon interaction point. This intrinsic uncertainty is also related to the positron range, photon noncollinearity, [9], and the tomographic reconstruction algorithm [10] which are the limiting factors of the spatial resolution of PET and PEM systems [11]. For conventional PET scanners the DOI effect is limited by using a relatively large detector diameter and a FOV close to the system center.

In breast PET cameras, it is essential to achieve an optimum trade-off between sensitivity and spatial resolution. Therefore the main challenge for the novel cameras is to improve the sensitivity without degrading the spatial resolution. Due to this, to increase the sensitivity by arranging the detectors closer to the breast, the DOI has to be measured and then its effects corrected, avoiding mis-positioning errors that decrease the spatial resolution. Recently, several studies described a number of detector designs with DOI information and different correction methods [12–15]. In general, when the photons reach the detector surface with small angles of incidence or close to the perpendicular direction, the DOI effects can be accurately corrected. By contrast, when the angle of incident is higher and the photons reach the detector surface close to the parallel direction, the DOI correction is often less accurate.

In last years, an increasing number of PEM prototypes and commercial systems were proposed [16–19] using very different detector geometries. Although all proposed PEM scanners offer significantly higher sensitivity than conventional PET, different performance between the proposed PEM scanner geometries can be found. In general, a PEM scanner consists of a bed and a gantry supporting the detectors. Several PEM scanners can be defined based on the arrangements of panel detectors. Firstly, scanners can have four panel detectors (in a box configuration) or only two detectors (dual head). A second class of PET scanners can include polygonal or even ring arrangements of several panel detectors which are fixed cameras, that is, the rotation is not necessary to acquire all angles.

The understanding of the properties of the detection systems used in PEM systems is essential for establishing appropriate operating criteria or designing schemes. In PET and PEM, two gamma photons (511 keV) from a positron-electron annihilation process are detected by means of a scintillator material. This material involves the conversion of the photon pair into visible light. Due to that the scintillator is optically coupled to a photomultiplier tube (PMT) the visible light can be converted to an electrical signal. This information is used to compute the spatial location of the photon interactions (photon interaction point) and the total energy deposited. When two photons from the same annihilation are detected in time coincidence then a line-of-response (LOR) can be defined and an event useful for the tomographic reconstruction is recorded. Nevertheless, some photons may escape after depositing only part of their energy into the crystal or even without interacting so that the event may be lost. The probability that a photon is detected depends on the scintillation material used and the crystal thickness.

Several authors have investigated the relation between the performance of a PEM scanner and its detector geometry configuration. Thus, Moses and Qi [20] carried out a comparison between the most common geometry based on a pair of parallel detector planes (dual head) and a rectangular box detector configuration. The results showed that the box geometry encircling the breast had better performance than the dual head detector as long as the DOI effect is compensated. More recently, Habte et al. [21] studied in detail the performance of different detector configurations using Monte Carlo simulation. Simulation results showed that the best performance was found for a PET scanner built from detectors arranged into a box-shaped geometry. The sensitivity for the box detector geometry was even higher than other geometries based on polygonal detectors encircling the breast. This is because when the detectors are arranged into a cylindrical system a significant number of intermodule gaps is produced by the rectangular-shaped detectors. This gaps provide a path for some photons escape. 

In this paper, we carry out an analytical study of the effect of different detector geometries on the photon sensitivity and on the angle of incidence of the detected photons which is related to the DOI effect. To this end, dual-head detectors are compared to box and different polygon detector configurations including rectangular parallelepiped crystals filling the intermodule gaps in order to avoid the drawback of these scanner geometries.

## 2.   Material and Methods

### 2.1. Detection Systems

**Figure 1:**
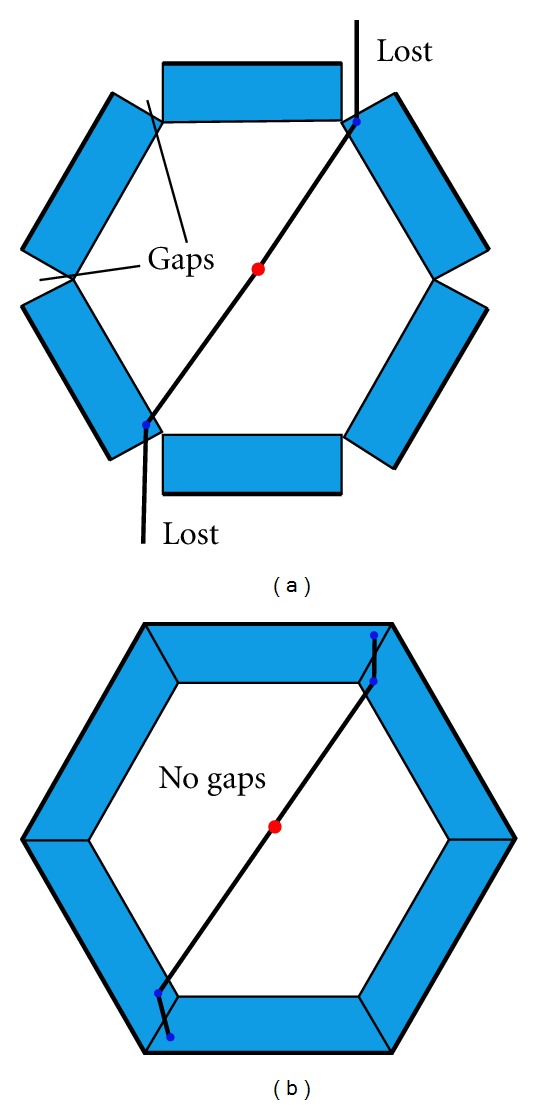
Effect of intermodule wedge-shaped gaps (a) with respect to a camera with filled gaps (b).

The most common PEM scanner geometry based on dual head detectors was compared to other geometries encircling the breast that included box and polygonal arrangements of panel detectors. As reported by Habte et al. [21], the drawback of these scanner geometries based on detectors formed into a cylindrical system configuration is that produce gaps which a decrease of the system sensitivity. In this regard, the gaps effect is increased when the number of detector modules of the scanner configuration is increased and it is lower for box configuration than for other detector configuration based on polygons of more sides. In order to avoid the gaps effect, we use rectangular parallelepiped detector modules filling the inter-module gaps. As it is shown in Figure 1 the use of these crystals can fill in the gaps with additional material that allows the detection of Compton-scattered photons.

**Figure 2:**
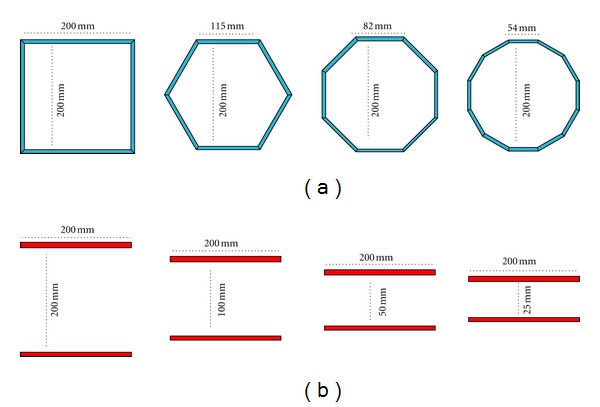
Different arrangements of panel detectors for a PEM scanner. They are based on polygons (a) and different two detectors schemes (b).

Figure 2 shows the different arrangements based on polygons (hexagon, octagon and dodecagon), four detectors (box) and two detectors considered for our purpose. Polygonal and box configurations are built by using parallelepiped detector modules. The distance between opposing detectors is 200 mm. 

The configuration based on two detectors makes possible to decrease this distance and therefore distances of 200 mm, 100 mm, 50 mm, and 25 mm are also considered.

### 2.2. Physical Performance of PET Scanners

The physical performance of a PET scanner can be studied by using different parameters.

#### 2.2.1. Sensitivity

Sensitivity of a PET scanner is defined as the rate in counts per second between detected true coincidence events and a given source activity. It depends on the material used as scintillator crystal, the geometry of the arrangements of the detectors, the energy threshold, and the time coincidence window.

#### 2.2.2. Uniformity

Uniformity is defined as the maximum relative deviation of counts obtained from an acquisition by using an extended uniform source. It depends on multiple factors such as PMT performance, inhomogeneities of the scintillator crystal, or changes in sensitivity along the FOV.

#### 2.2.3. Spatial Resolution

Spatial resolution of a PET scanner is defined as its ability to distinguish between two points after image reconstruction, that is, it is the distance between adjacent detection points. The spatial resolution can be characterized by the full width half maximum (FWHM) in mm of the image of a point source in air. It depends on the interaction point estimation (intrinsic spatial resolution) and the tomographic reconstruction algorithm.

### 2.3. Analytical Estimation of Photon Sensitivity

The photon sensitivity of a PET system is determined by the intrinsic efficiency (*E*_*i*_) that is related to the detector material, the geometric efficiency (*E*_*g*_) that is related to the detector configuration, and the threshold (*E*_th_) related to the energy and coincidence windows:

(1) $$\begin{array}{c} S_{\text{PET}}=E_{i}\cdot E_{g}\cdot E_{\text{th}}. \end{array}$$

#### 2.3.1. Intrinsic Efficiency

*E*_*i*_ is the average intrinsic photon stopping efficiency and it is defined as the probability that two annihilation photons traversing the detector material are absorbed. It is given as the squared of the single efficiency of detecting each photon (*E*_*i*1_ and *E*_*i*2_):

(2) $$\begin{array}{c} E_{i}=E_{i1}\cdot E_{i2}=\left(1-e^{-\mu (E_{1})\cdot x_{1}}\right)\cdot \left(1-e^{-\mu (E_{2})\cdot x_{2}}\right), \end{array}$$

where *x*_1_ (and *x*_2_) is the thickness of the crystal traversed along the incident line of each photon. It depends on the angle of incidence *θ* of the each incoming photon: for perpendicular photon incidence *x*_1_ is equal to the detector thickness; for all other photon incidences (*θ* > 0) it is higher than the detector thickness. Finally, *μ*(*E*_1_) (and *μ*(*E*_2_)) is the total linear attenuation coefficient (photoelectric and Compton scatter) of the crystal material at the each incoming photon energy. It depends on the photon energy but also on the density and atomic number of the detector material.

**Figure 3:**
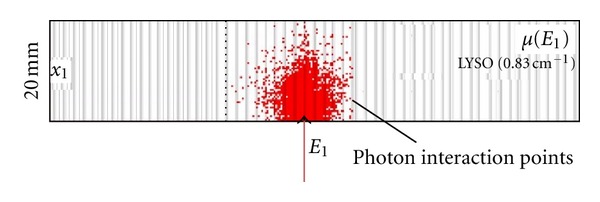
Intrinsic efficiency.

Figure 3 shows the distribution of photon interaction points of a high number of incoming photons of 511 keV for LYSO crystal blocks of 200 × 200 × 20 mm^3^ which are considered for our evaluation. This material possesses excellent characteristics for detecting 511 keV photons such as the atomic number (*Z* = 65), the density (*d* = 7.1 g/cm^3^), and the attenuation coefficient is (*μ* = 0.83 cm^−1^).

#### 2.3.2. Geometric Efficiency

**Figure 4:**
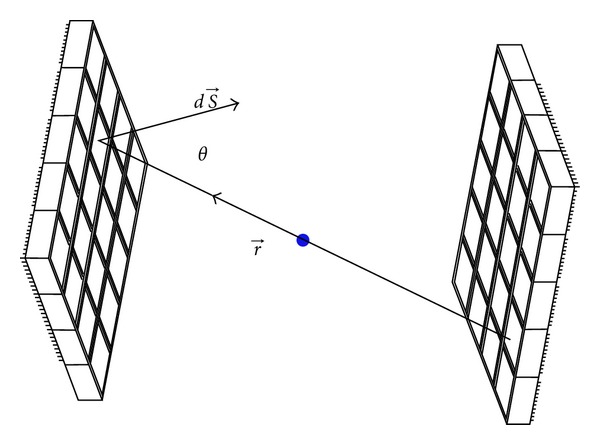
Analytical estimation of the geometric efficiency.

*E*_*g*_ is the total solid angle coverage of the detectors and it is defined as the probability that two annihilation photons intercept the detector area. The total solid angle fractional coverage (*Ω*) of the system is given as follows:

(3) $$\begin{array}{c} E_{g}=\frac{\Omega }{4\pi }=\frac{\iint \vec{r}\cdot d\vec{S}}{4\pi \pi^{2}}, \end{array}$$

where *r* is the distance from the image point to the detector bin (higher efficiency is obtained for image points placed close to the detector) and *dS* is the surface normal vector of an infinitesimal area of the detector (Figure 4).

An estimation of the total solid angle (*Ω*) can be obtained by dividing the area of each detector into finite detector elements so that *E*_*g*_ is calculated as follows:

(4) $$\begin{array}{c} E_{g}=\sum_{i=1}^{i=n\;\text{bins}}\frac{\cos\left(\theta \theta \cdot \Delta S\right)}{4\pi \pi^{2}}, \end{array}$$

where *θ* is the angle of incidence (*θ* = 0° corresponds to an incident photon perpendicular to the detector surface and *θ* = 90° to an incident photon parallel to the detector surface) and Δ*S* is the area of the finite detector element.

### 2.4. Angle of Incidence

**Figure 5:**
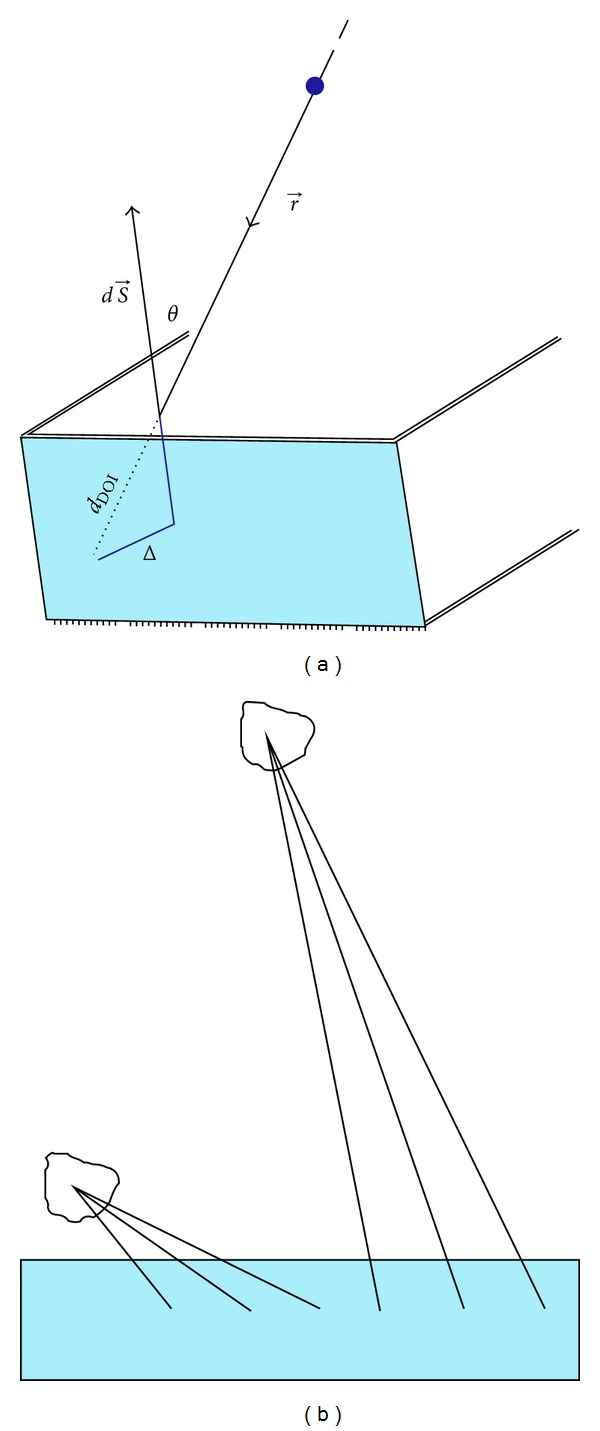
DOI and mis-positioning (Δ) in photon interaction point (a). This effect increases with the proximity of the detectors to the breast, due to that the angle of incidence is increased (b).

As can be observed in Figure 5, many photons penetrate a significant distance into the detectors before they are detected. This distance is called depth of interaction (DOI). Due to the proximity of the detectors to the breast the angle of incidence *θ* can be very high and therefore this effect can cause a deterioration of the intrinsic spatial resolution.

If the incoming photon direction or angle of incidence is *θ* and the DOI is *d*_DOI_ then the mis-positioning of the photon interaction point with respect to the real position is estimated as follows:

(5) $$\begin{array}{c} \Delta =d_{\text{DOI}}\cdot \sin\left(\theta \right). \end{array}$$

The mis-positioning originated from the DOI effect is obtained for the different detector configurations in order to evaluate the need of accurate methods to compensate this effect.

## 3. Results and Discussion

### 3.1. Photon Sensitivity

The photon sensitivity at a centered point was obtained for the different detector arrangements and it is shown in Table 1. The different PEM scanner geometries considered were dual, box, hexagon, octagon, and dodecagon configurations with a distance of 200 mm between opposing detectors such as shown in Figure 2. The photon sensitivities were obtained without considering the threshold (*E*_th_) related to the energy and coincidence windows. As expected, higher sensitivity was obtained for box and the different polygon detectors compared to the sensitivity of dual detector configuration. This is because the dual detector configuration does not completely close the ring around the object. No significant differences were found between the box detector and the different polygon detector configurations. This is because of the fact that there are not intermodule gaps as in the results shown by Habte et al. [21] and therefore the only differences between box and polygon configurations come from the term in *E*_*g*_ formula related to the angle of incidence.

Although the dual head configuration showed a lower sensitivity than the polygon configurations it has the advantage that the distance between opposing detectors can be decreased in order to adapt it to the object size. This is particularly interesting due to the variable size of breast in women. Thus, the distance between opposing detector can be decreased for small breast. This is clearly an advantage of dual head cameras with respect to fixed cameras.  Table 2 shows that the sensitivity at a centered point increases when the distance between opposing detectors is decreased.

### 3.2. Uniformity

**Figure 6:**
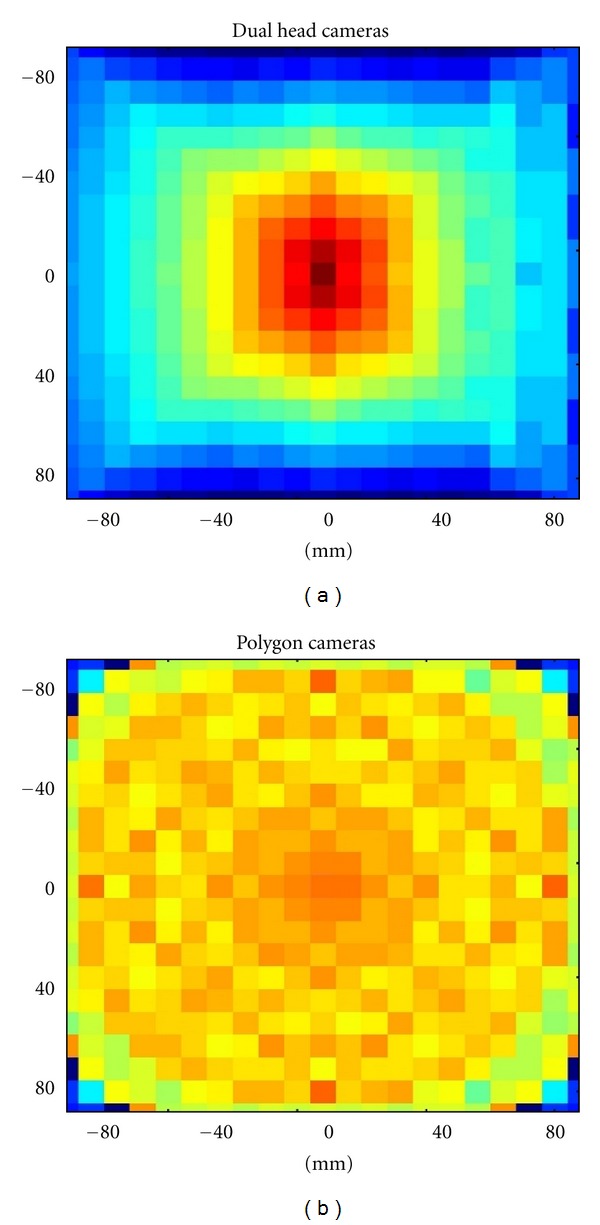
Sensitivity along the FOV for dual-head cameras (a) and polygon cameras (b).

**Figure 7:**
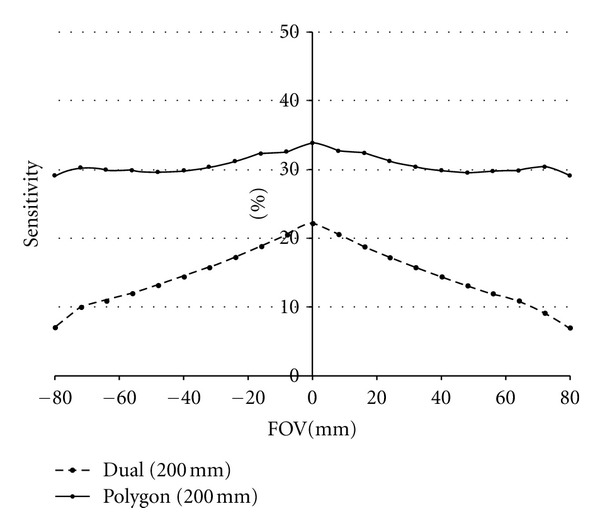
Transverse profiles of sensitivity for dual-head cameras (left) and polygon cameras (right).

Figure 6 shows the uniformity in terms of the sensitivity changes along the FOV for dual head and polygon (dodecagon) cameras. These sensitivity images were obtained for a distance of 200 mm between opposing detectors such as shown in Figure 2. The sensitivity is clearly more uniform for polygon configuration compared to the sensitivity for dual-head cameras. This means that the signal-to-noise ratio in the reconstructed image for polygon scanners will be similar for each point of the FOV. Nevertheless the signal-to-noise ratio will be higher for central points and decreases towards the limits of the FOV for dual-head cameras. This different behaviour can be seen in Figure 7 that shows the transverse sensitivity profile along the FOV for dual-head and polygon cameras. 

### 3.3. DOI and Intrinsic Spatial Resolution

The averaged mis-positioning of the photon interaction point due to the DOI effect at a centered point is shown in Table 3 for the different scanner geometries. For the dual detector configuration the mis-positioning increased when the distance between opposing detector is decreased. For the box detector configuration the mis-positioning was slightly lower than for the different polygon detectors. No significant differences were found between the various polygon detectors. The averaged mis-positioning values at the edge of the FOV were lower than the mis-positioning values at the center of the FOV for all geometries but for the box configuration. Very high maximum mis-positioning values were found for all geometries at center and edge of the FOV. 

These results show a significant effect of the DOI on the mis-positioning of the photon interaction point and therefore on the intrinsic spatial resolution for all detector geometries. Furthermore, this becomes an essential issue to achieve a high performance of the scanner since this effect is greater at the center than at the edge of the FOV.

## 4. Conclusions

An analytical study of the performance of different PEM detector geometries in terms of photon sensitivity and DOI effect was carried out in order to find an optimal arrangement of the detectors. To this end, dual-head detectors were compared to box and different polygon detector configurations.

Our results showed that higher sensitivity and uniformity are obtained for box and polygon detector configurations compared to dual-head cameras. For the polygon configurations the sensitivity is only moderately increased when the number of detectors is raised. The variable size of breast in women is an advantage for dual-head cameras with respect to to fixed cameras. Thus, for dual head cameras the sensitivity can be increased for small breasts by decreasing the distance between opposing detectors. Nevertheless this translates in an increase of the mis-positioning of the photon interaction point due to the DOI effect. 

The optimal configuration in terms of sensitivity is a PEM scanner based on a polygon of twelve (dodecagon) or more detectors. We have shown that this configuration is clearly superior to dual-head detectors and slightly higher than box, octagon, and hexagon detectors. Nevertheless, DOI effects are increased for this configuration compared to dual-head and box scanners and therefore an accurate compensation for this effect is required. 

## Figures and Tables

**Table 1:** Sensitivity at a centered point for a distance of 200 mm between opposing detectors.

Configuration	Sensitivity at center
Dual detector	8.4%
Box detector	33.3%
Hexagon detector	34.0%
Octagon detector	34.2%
Dodecagon detector	34.5%

**Table 2:** Sensitivity at a centered point for dual head cameras for different distances between opposing detectors.

Distance (FOV)	Sensitivity at center
200 mm	8.4%
100 mm	14.8%
50 mm	19.6%
25 mm	22.2%

**Table 3:** Mis-positioning of the photon interaction point due to the DOI effect at center and edge (80 mm) of the FOV.

Configuration	FOV (mm)	Mis-positioning center FOV (mm)		Mis-positioning edge FOV (mm)	
		Averaged	Maximum	Averaged	Maximum
	200	2.9	8.1	1.9	5.8
Dual detector	100	4.0	9.4	2.6	6.7
	50	4.6	9.8	3.0	7.0
	25	4.8	10.0	3.1	7.1
Box detector	200	3.8	8.1	4.1	9.7
Hexagon detector	200	5.0	7.5	2.9	9.2
Octagon detector	200	4.7	7.3	2.5	9.3
Dodecagon detector	200	4.4	7.2	1.7	9.3
